# Chicago Medical Society

**Published:** 1885-04

**Authors:** 


*Meeting of February 16, 1885.*—The President, Dr. D. A. K. Steele, in the chair.

*Malignant Disease of the Thyroid Gland.*—Dr. C. E. Webster reported the case of a woman, sixty years old, whose general health was quite good, but who first noticed an enlargement of her neck about a year before she first consulted him. It began in one lobe of the thyroid, and extended rapidly to the adjacent glands and tissues of the neck. When she was first seen, swallowing was difficult and her voice was husky, although her breathing was not impeded. The thyroid gland, the larynx, the trachea, and the neighboring cervical glands formed an irregular doughy mass. The diagnosis lay between tertiary syphilis and cancer of the thyroid, but it was made easy by the history of the progress of the growth from an enlargement of the thyroid, and by the fact that such growths after middle life were almost invariably malignant. The steady progress of the disease under a short course of anti-syphilitic treatment confirmed the diagnosis, and the patient had recently died of exhaustion. The microscopical appearances of such growths were sometimes peculiar, hardly differing from those of benign tumors of the same organ, so that it was often difficult to decide upon the nature of the neoplasm from those appearances alone. A case in point was cited from the service of the Massachusetts General Hospital, in which the alveoli were lined with cuboid cells and filled with a homogeneous substance.

Dr. F. Cary asked where the cells were in the latter case, if the alveoli were filled with a homogeneous substance.

Dr. L. H. Montgomery asked about the dimensions of the tumor, whether it extended uniformly in all directions, and if the speaker had any theory as to the functions of the thyroid. Might not the organ be situated over the trachea for purposes of protection, or did it act as a sort of reservoir, like the spleen?

Dr. E. J. Doering asked if removal of the mass would have been justifiable.

Dr. Webster stated that, in the hospital case referred to, the lumen of each alveolus was filled with a homogeneous substance, and the cells were arranged peripherally. When he first saw his own patient the disease had progressed to such a stage that, had an operation been undertaken, it would have been impossible to complete it, as the deep tissues of the neck, including the œsophagus, were involved in the growth. The tumor of the thyroid was as large as half a hen's egg, and a doughy mass extended in all directions. He had no theory to offer as to the function of the thyroid gland.

*Tape-Worm in a Child.*—Dr. C. G. Davis read a report of a case of *Tœnia solium* in a child two years old, and showed about four inches of the worm, including the head. On the 10th of December, when he first saw the child, it had not quite recovered from an attack of entero-colitis that had lasted through the summer and autumn; it still occasionally had the usual symptoms of "summer complaint." A number of simple remedies were given, together with raw beef, and this treatment seemed to be effectual, but portions of tænia soon began to appear in the discharges, and half a teaspoonful of a French preparation of pelletierine was given, followed in an hour by twenty drops of tincture of jalap and a teaspoonful of castor-oil. This was followed by the expulsion of three or four yards of the worm, but the head was missing. The child was then carefully nursed, and its general health looked after, when segments of the worm again appeared in the evacuations. A double dose of the pelletierine, jalap and castor-oil was then given, and several feet more of the worm were discharged, including the head, which was now exhibited.

Dr. Doering alluded to three cases of tænia that he had treated in children, the youngest of whom was only six months old and had been fed on raw beef while sick with entero-colitis. He was surprised at the results that had followed the treatment in Dr. Davis's case, for he himself had used much larger doses of pelletierine, with only partial success.

*Hygroma of the Tongue.*—Dr. Josef Zeisler related the case of a girl, nine years old, the right side of whose face was much more developed in its osseous and muscular formation than the left side. The surface of the tongue was covered with cysts, varying in size from that of a pin-head to that of a pea, and there was a collection of like vesicles on the mucous membrane of the right cheek, near the angle of the mouth. There was also a crested formation running along the middle line of the dorsum of the tongue, of a caruncular appearance. The movements of the tongue and the faculty of speech were not impaired. The speaker regarded the case as one of colloid degeneration of the mucous membrane, but he could not classify it precisely, having never seen another like it. The only treatment suggested was galvano-puncture of the cysts. A microscopical examination was not allowed. Dr. E. Andrews, Dr. C. T. Parkes, Dr. J. N. Hyde, and several other well-known physicians had seen the patient, but none of them had seen a case of the sort, and they could make no further suggestion in regard to treatment. Two like cases were to be found in the literature of the subject.

*The Comma Bacillus.*—Dr. L. L. McArthur showed specimens of Koch's cholera bacillus. He considered that, thus far, Koch had successfully met all opposition to his theory of the causal relation of the bacillus to Asiatic cholera. The specimens shown were from Koch's laboratory, having been sent by Dr. Betz, of Tübingen, to Dr. Doering.

